# High-Throughput Spike Detection in Greenhouse Cultivated Grain Crops with Attention Mechanisms-Based Deep Learning Models

**DOI:** 10.34133/plantphenomics.0155

**Published:** 2024-03-11

**Authors:** Sajid Ullah, Klára Panzarová, Martin Trtílek, Matej Lexa, Vojtěch Máčala, Kerstin Neumann, Thomas Altmann, Jan Hejátko, Markéta Pernisová, Evgeny Gladilin

**Affiliations:** ^1^ Mendel Centre for Plant Genomics and Proteomics, Central European Institute of Technology (CEITEC), Masaryk University, Brno, Czech Republic.; ^2^ National Centre for Biomolecular Research, Faculty of Science, Masaryk University, Brno, Czech Republic.; ^3^ Photon Systems Instruments, spol. s r.o., Drasov, Czech Republic.; ^4^ Faculty of Informatics, Masaryk University, Botanicka 68a, Brno, Czech Republic.; ^5^ Leibniz Institute of Plant Genetics and Crop Plant Research, Seeland OT Gatersleben, Germany.

## Abstract

Detection of spikes is the first important step toward image-based quantitative assessment of crop yield. However, spikes of grain plants occupy only a tiny fraction of the image area and often emerge in the middle of the mass of plant leaves that exhibit similar colors to spike regions. Consequently, accurate detection of grain spikes renders, in general, a non-trivial task even for advanced, state-of-the-art deep neural networks (DNNs). To improve pattern detection in spikes, we propose architectural changes to Faster-RCNN (FRCNN) by reducing feature extraction layers and introducing a global attention module. The performance of our extended FRCNN-A vs. conventional FRCNN was compared on images of different European wheat cultivars, including “difficult” bushy phenotypes from 2 different phenotyping facilities and optical setups. Our experimental results show that introduced architectural adaptations in FRCNN-A helped to improve spike detection accuracy in inner regions. The mean average precision (mAP) of FRCNN and FRCNN-A on inner spikes is 76.0% and 81.0%, respectively, while on the state-of-the-art detection DNNs, Swin Transformer mAP is 83.0%. As a lightweight network, FRCNN-A is faster than FRCNN and Swin Transformer on both baseline and augmented training datasets. On the FastGAN augmented dataset, FRCNN achieved a mAP of 84.24%, FRCNN-A attained a mAP of 85.0%, and the Swin Transformer achieved a mAP of 89.45%. The increase in mAP of DNNs on the augmented datasets is proportional to the amount of the IPK original and augmented images. Overall, this study indicates a superior performance of attention mechanisms-based deep learning models in detecting small and subtle features of grain spikes.

## Introduction

Precise and dependable extraction of phenotypic traits from extensive image data is essential for studying the impact of biotic and abiotic factors on cereal crops’ yield and agronomic robustness. Detecting spikes in cereal crops within the greenhouse environment poses a great challenge due to the varying optical settings, illumination conditions, and spike appearance observed in greenhouse images obtained from different phenotyping facilities [1]. In particular, the detection of emergent spikes in the inner plant regions is hampered by the similarity of spike and leaf colors and the lack of explicit spike contours in European bushy wheat cultivars. To address the problem of small-spike pattern detection, one can increase the feature resolution by simply magnifying the input images. However, this results in significantly higher time consumption for deep neural network (DNN) model training and testing and loss of rich contextual features of the spike in the pre-processing stage. An alternative approach consists of increasing an object’s feature dimension, which can be achieved by combining the enhanced high-level features and lower-level features, thus generating a single multiscale feature representation of the object [2]. However, the extracted feature may not be discriminative and differentiable enough for localizing small inner spikes. Bi et al. [3] developed various architectures for a 3-layer neural network, adapting the number of hidden layer nodes to classify 4 distinct wheat varieties to extract spike traits. Misra et al. [4] introduced SpikeSegNet, a system that accomplishes spike detection through the utilization of 2 successive feature networks: one dedicated to local patch extraction and the other focused on global mask refinement. Pound et al. [5] deployed a DNN designed for the recognition of spikelets and subsequent counting.

**Fig. 1.**
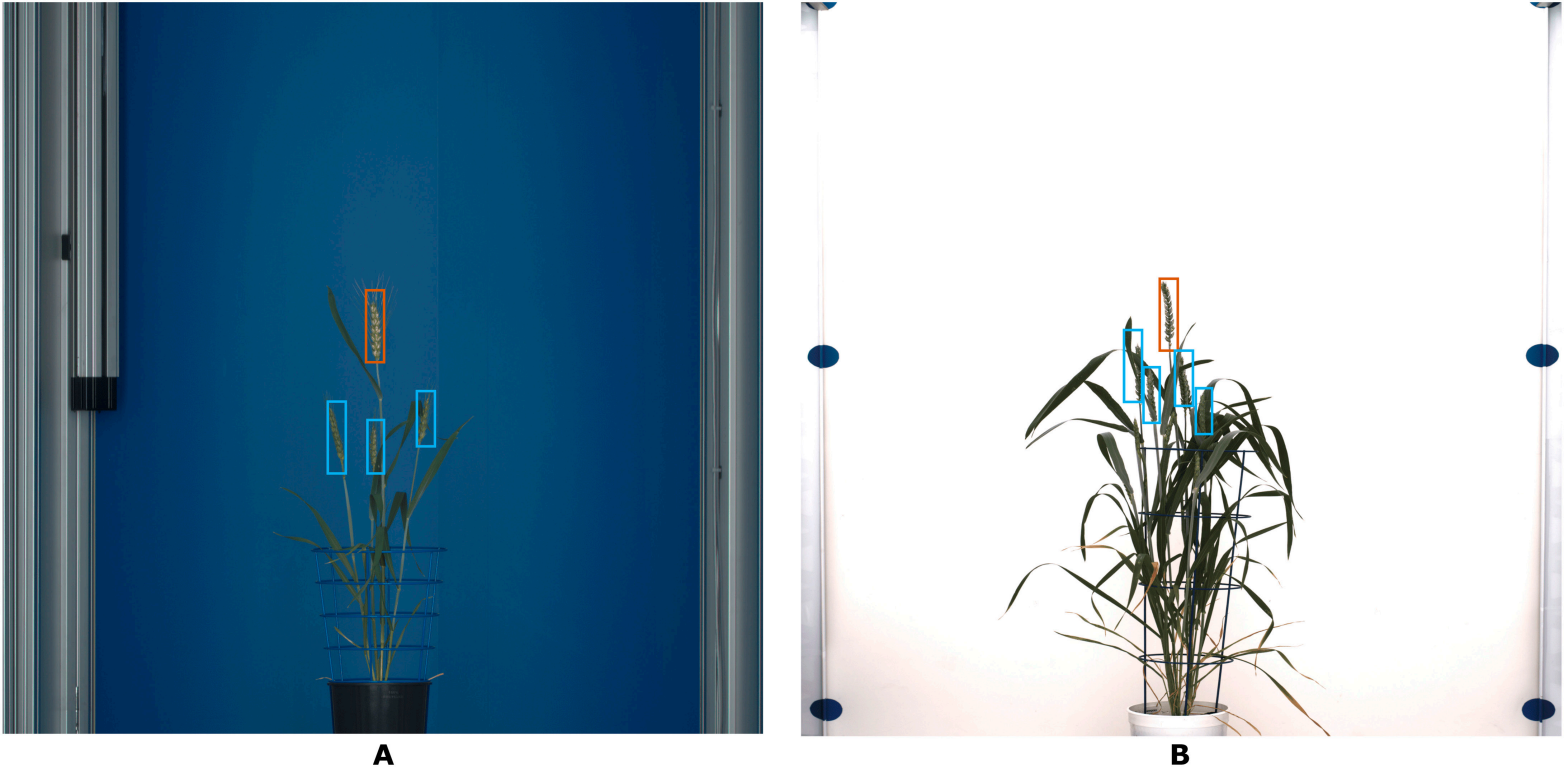
Examples of side view images of wheat cultivars from 2 different greenhouse phenotyping facilities: (A) PSI and (B) IPK. Top spikes are enclosed in orange boxes, while inner spikes are enclosed in light blue boxes.

Qiongyan et al. [6] segmented the top spikes in wheat by extracting features with Frangi filters and subsequently training them as input to artificial neural networks (ANNs). Narisetti et al. introduced various enhancements to the shallow ANN architecture, including integrating Frangi line filters, which could significantly improve the final segmentation results. Nevertheless, further significant efforts are still required within the ANN framework, particularly in terms of parameter tuning for newly acquired greenhouse images [7]. Mottaghi et al. [8] designed a novel deformable part-based model that exploits global and local contexts around each candidate detection, which can help detect objects at all scales, especially in the case of small objects. Using R-CNN, Hasan et al. [9] accomplished the spike counting and detection task, achieving an impressive F1 score of 0.95 on a dataset comprising 20 wheat field images, with a mean spike count between 70 and 80 per image. In recent years, several improvements have been proposed to RCNN [10] and Faster-RCNN (FRCNN) [11]. He et al. [12] proposed improvement in RCNN by adding a pyramid pooling layer before the convolution layer on the feature map. One of the benchmark datasets, PASCAL Visual Object Classes (VOC), which contains 20 object categories, is the most widely used benchmark dataset for generic object detection. The object instances in the PASCAL VOC occupy a large pixel area compared to the entire image and other benchmark datasets, such as the COCO dataset of similar nature. Current object detectors, like FRCNN, always leverage the convolutional neural networks to extract increasingly more abstract feature representations. During this process, the intermediate feature maps of small-scale objects are usually down-sampled too often by the convolutional or pooling layers, whose stride is greater than one. In FRCNN, when trained on small-scale objects, the prominent extracted and salient features do not have enough information in the feature map to make accurate localization tasks. Therefore, it is unsurprising that direct application of conventional FRCNN is associated with reduced accuracy by detecting small patterns such as spikes [11,13]. Consequently, in this work, we introduced some modifications to the conventional FRCNN architecture that mitigate the loss of feature information, such as the number of convolutional layers, filter combination with an added attention mechanism to retain, and then enhance the relevant high-value features of mixed wheat cultivars of greenhouse spikes. In our previous research [1], the 2 most prominent DNN frameworks for pattern detection, FRCNN and YOLOv3/v4, were evaluated on the detection of spikes from 2 different phenotyping facilities (further referred to as Photon Systems Instruments [PSI] and Leibniz Institute of Plant Genetics and Crop Plant Research [IPK] datasets, respectively) (see Fig. 1). Thereby, both DNN detection models were trained on ground truth images from only one facility (PSI) but evaluated on both PSI and IPK images.

The prediction of both detectors performed well on PSI data with spike detecting and evaluating measure, mean average precision (mAP) with a range between 0.78 ≤ *mAP*_0.50_ ≤ 0.95 and 0.47 ≤ *mAP*_0.50−0.95_ ≤ 0.66 on wheat cultivars, depending on different spike locations [1]. Both *mAP* are defined in the “Evaluation metric in detection DNNs” section. With FRCNN (on *mAP*_0.50_), the mAP of the top spike was 0.99, and the inner spike was 0.91 (8.08% mAP drop) on the PSI wheat cultivar. However, when applied to “unseen” (external validation set) images of another greenhouse setup (IPK), both DNN detectors showed a significantly lower accuracy of spike pattern detection (0.233 ≤ *mAP* ≤ 0.410). In this work, we dealt with improving the accuracy and the generalizability of the FRCNN detection framework by applying it to previous images from different optical setups. In contemporary literature, the acquired wheat dataset from the phenotyping facilities contains single or multiple cultivars whose spikes have similar optical appearance and texture, and the trained DNNs are used to predict that particular dataset [6,7,9]. Retraining DNNs for each new cultivar image data is quite demanding and inefficient. Therefore, it is worthwhile to aid the model performance with regularization techniques to diversify the representative dataset with more image augmentation strategies to make robust DNNs. The diversity of images should be enough for the model to learn the deviation from normal spike appearance and also handle, in some instances, noisy targets without hampering the model’s accuracy. Since the generation of ground truth data for DNN model training is the main bottleneck of the entire DNN training pipeline, our study addresses important questions of accuracy of pattern detection cross-application to new “unseen” data and strategies of its adaptation utilizing data augmentation. In augmentation strategies such as generative adversarial network (GAN), firstly, the study of the learning from augmented spike image is necessary to determine whether DNNs learn from GAN-augmented and retain their performance over the original image. Secondly, to find out the right proportion of retraining image of each cultivar when they have a different colormap with respect to the background, it is useful to not compromise on accuracy and keep overhead at a minimum.

**Fig. 2.**
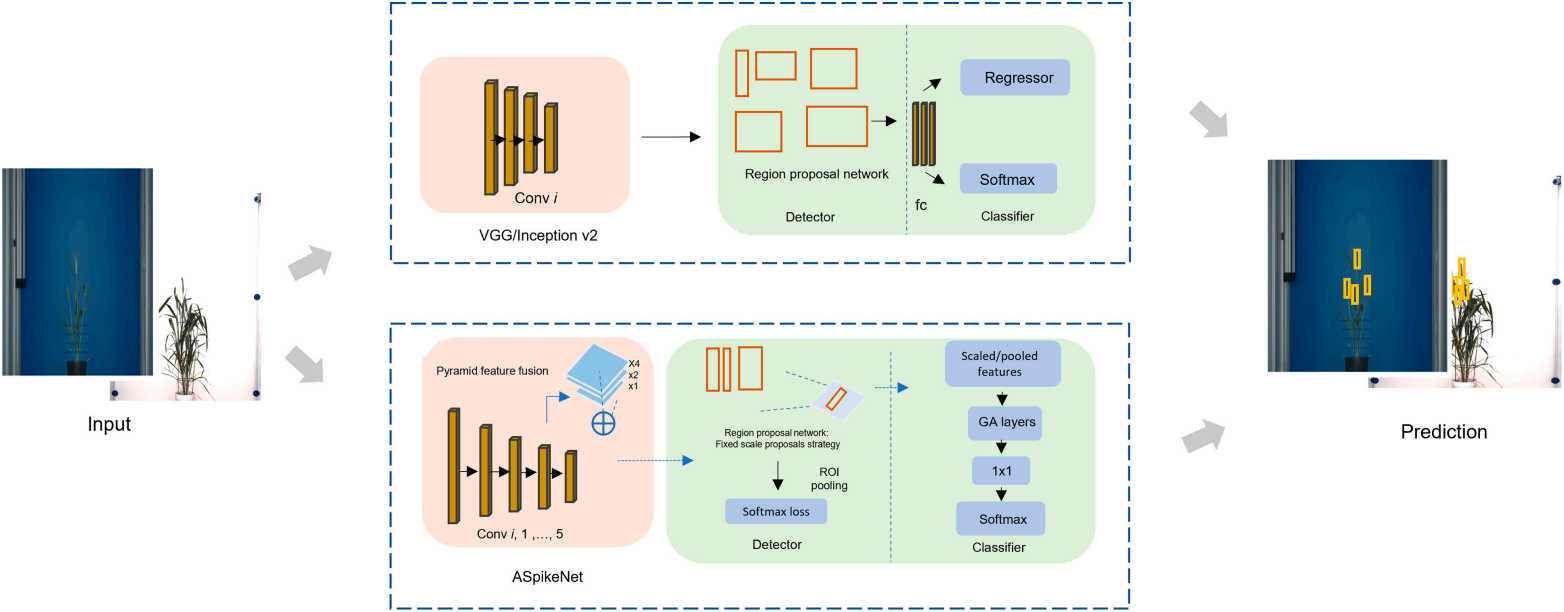
Comparative schemes of the training process and elements of the architecture of FRCNN (top) vs. FRCNN-A (bottom).

Recently, deep learning models have incorporated attention mechanisms in machine learning applications to extract the noticeable features of an object in convolution layers, give weightage to the salient features, and disregard the less prominent features. Hu et al. [14] proposed their Squeeze-and-Excitation (SE)-Net, an attention module that computes the channel dimension of the feature map in each layer and thus gives weights to some prominent feature of an object and subdues the rest of the dormant features. Guo et al. [15] proposed SPA-Net, which introduces spatial pyramid structure into a single attention module. It combines structural regularization of objects and also structural information. Hu et al. [16] proposed a competitive squeeze-excitation mechanism that captures the competitive relationship from both the residual and identity channel and extends the channel-wise attention module. The transformer-based models are state-of-the-art detection models that employ a self-attention mechanism [17,18]. In this study, we adapted the attention mechanism in convolution layers of FRCNN to capture the feature map of spikes. The general pipeline of FRCNN and FRCNN-A is depicted in Fig. 2. Our contribution to this work can be summarized as follows:

1. We proposed ASpikeNet, a residual network reducing the non-essential convolutional layers in the backbone architecture and adapting to localize the small-scale object such as spike.

2. We improved network robustness with an attention mechanism in FRCNN-A to inhibit the false-positive prediction, primarily the inner spikes in the plant.

3. We conducted a comparative analysis of FRCNN and FRCNN-A with a state-of-the-art Swin Transformer and evaluated their performance on spatially classified spikes.

## Materials and Methods

### Image data

In this study, visible light images of greenhouse-grown wheat cultivars from IPK (Gatersleben, Germany) and PSI (Drásov, Czech Republic) were acquired. Building upon our previous work [1], which utilized images of wheat from a single phenotyping facility, PSI, and number of cultivars for DNN model training, the current study expands the scope significantly. We have incorporated a new, more diverse dataset that includes images from 2 distinct locations: PSI and IPK. This dataset not only includes a greater variety of wheat cultivars (PSI:19 and IPK:122) but also enriches the training, validation, and augmentation processes of the DNN model. A baseline and the primary dataset were generated using greenhouse wheat images from IPK and PSI without any augmentation or transformation. From the IPK phenotyping facility, we acquired a dataset consisting of 3 distinct sets of images, each capturing several different angles from the side view of totally 150 randomly selected images of 122 unique wheat cultivars at the resolution of 3,315 × 4,462. The 3 distinct viewing angles correspond to 0 ^∘^ (52 images), 45 ^∘^ (43 images), and 90 ^∘^ (55 images). Only one viewing angle was used in acquiring 300 images from PSI. In the baseline dataset, 300 PSI and 150 IPK images were included. The annotation is crucial for training our machine learning models, as it provides them with the necessary information to learn and make accurate predictions. We used LabelImg software to make the ground truth around the spike [19]. The bounding boxes were drawn in a way so that it localized the spike and not all the elongated spike awns to ensure consistency between different cultivars. For training and testing purposes, the dataset was subdivided with the common ratio of 80:20, resulting in the training set of 360 (240 PSI and 120 IPK) and the testing set of 90 wheat images (60 PSI and 30 IPK), respectively.

### Experimental design

To test the robustness of DNN detectors on “unseen” cultivars and optical setups, 9 datasets, including PSI, IPK, and augmented IPK images, were generated for the training set (see Table 1). The test set consists of only original IPK images. The mixed composition of the original training set was used to test how far the FRCNN and our model trained on one particular dataset can be extended to another different wheat cultivar and optical setup by means of progressive data augmentation. The original dataset is stepwise extended with automatically generated new images from the new (target) phenotyping facility (in our case: IPK).

**Table 1.**
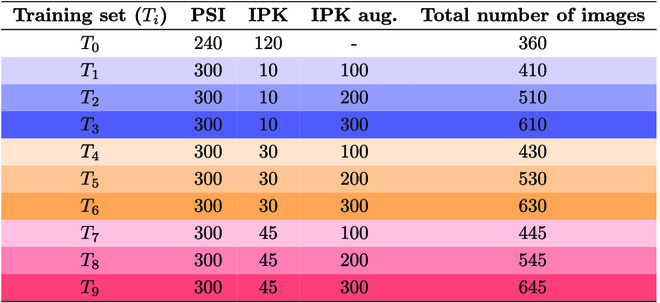
Overview of the 10 training image sets used for training of spike detection models that were generated by combining images from PSI (2022) and IPK (2018) greenhouse phenotyping facilities and progressively extended with automatically augmented IPK images. The color code corresponds to the relative amount of IPK images: blue gradient for *T*_1_ to *T*_3_, orange gradient for *T*_4_ to *T*_6_, and red gradient for *T*_7_ to *T*_9_, respectively.

**Fig. 3.**
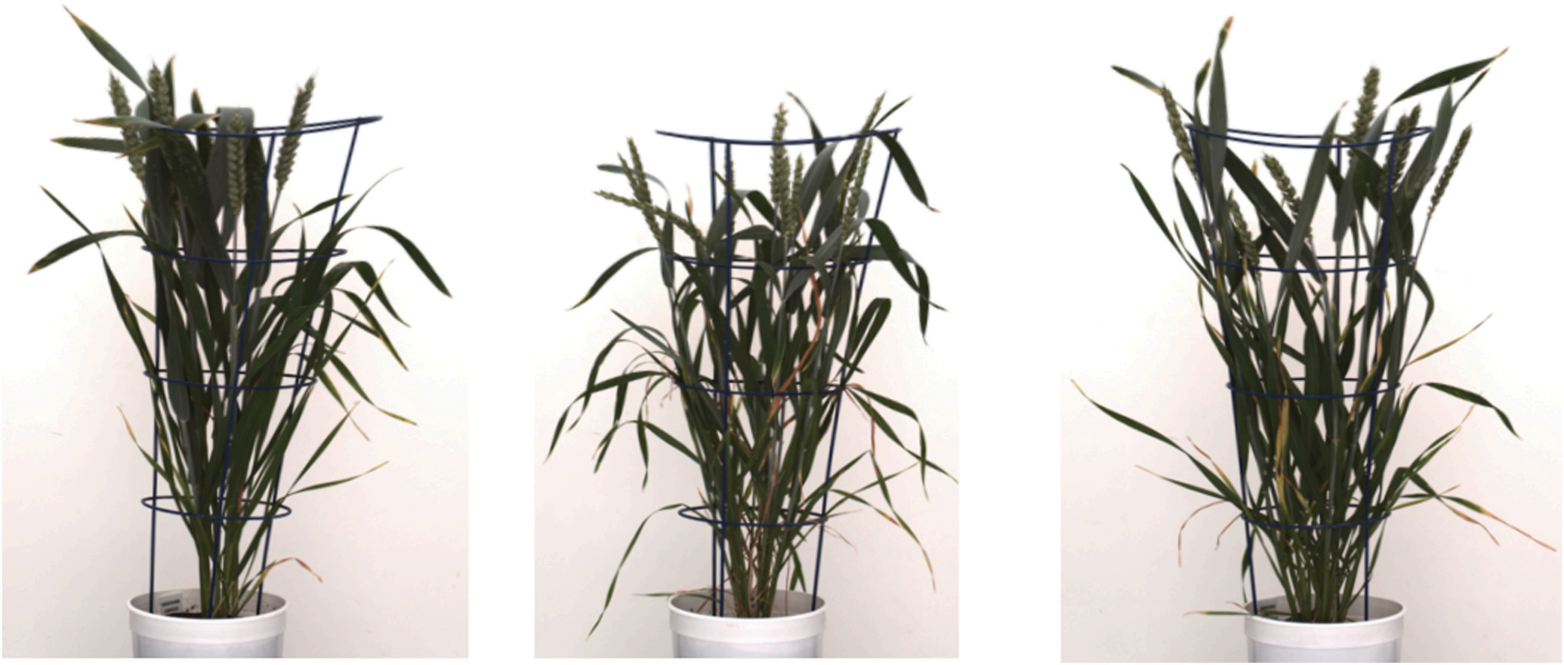
Examples of synthetic images of greenhouse-grown plants generated by FastGAN (selected from the epoch 80k).

The goal of these 9 IPK and IPK augmented image compositions aimed to dissect the performance of DNNs predominantly trained on PSI datasets that can be adapted to new images from IPK with minimum efforts on ground truth image generation. The strategy of conventional geometric transformation and the generative adversarial combination was used for data augmentation. In all the 9 compositions of the training sets, the IPK augmented images have the same proportion (50:50) from the geometric transformation and GAN-transformed images. To generate synthetic images for training, we implemented FastGAN architecture [20]. The model was trained in PyTorch with an input of resolution 1,024 × 1,024. The input for FastGAN is optimized for 1,024 × 1,024; therefore, we have cropped the right and left sides adjacent to the plant, and the region directly above the plant’s canopy, which is similar in all images. The average resolution of images after the cropping is 1,200 × 2,800. A total of 90k epochs were completed during the training phase. The generated images were taken from epoch 80k based on the Frechet inception distance (FID) and by choosing more realistic examples. The FID of the generated images is 157.50, calculated with PyTorch-fid library [21]. Figure 3 showcases a selection of examples generated by FastGAN.

### Spike detection DNN models

#### Faster RCNN

In our previous work [1], we used FRCNN with the Inception v2 backbone, fixed aspect ratio, and region proposal network (RPN) with 100 object candidates. This RPN largely optimized the aspect ratio for large objects such as PASCAL VOC. However, the fixed box areas of RPN are larger than the average spike bounding box (120 × 200). In our previous approach, the 3-aspect ratio used area are 0.5, 1, and 2 while the bounding boxes area are 128^2^, 256^2^ and 512^2^. Several modifications to FRCNN concerning the feature extractor, anchor box, and RPN are made to address this issue, described in the “FRCNN-A: Faster-RCNN with ASpikeNet and attention module” section. With an anchor box on either side, we add the average-size bounding box or choose the one that captures the feature resolution of the spikes. The architecture of FRCNN comprises feature extraction, region proposal, and the final detector. In the first stage, multiple objects are proposed as candidates for subsequent main object detectors. The object candidate is either spike or background. The input image has a resolution of 2,560 × 2,560. RPN uses *N* anchor boxes of 10 different aspect ratios and sizes at each location. RPN is initiated with zero mean Gaussian distribution. This anchor translation is invariant. It uses the same ones at every location. The addition of RPN attains higher accuracy but at the cost of computation resources. The feature in the convolution layer and main object detector is shared. In the first stage, 100 candidates are selected for feature extraction. They are labeled as either positive or negative based on their spike. The metric used to evaluate the selection of positive anchors is intersection over union (IoU). The threshold of IoU > 0.7 is used. The total loss is calculated as softmax loss (classification and L1 loss). As most anchors have the background and are negative (non-spike proposal), the loss is calculated in mini-batches to remove any bias. The features extracted in RPN are fed into region-of-interest (ROI) pooling as feature vectors for a fully connected layer. The output is getting at the binary output by the softmax layer with a set of class probabilities. The network is optimized by stochastic gradient descent (SGD), and exponential decay is used as a learning parameter.

#### Swin Transformer

**Fig. 4.**
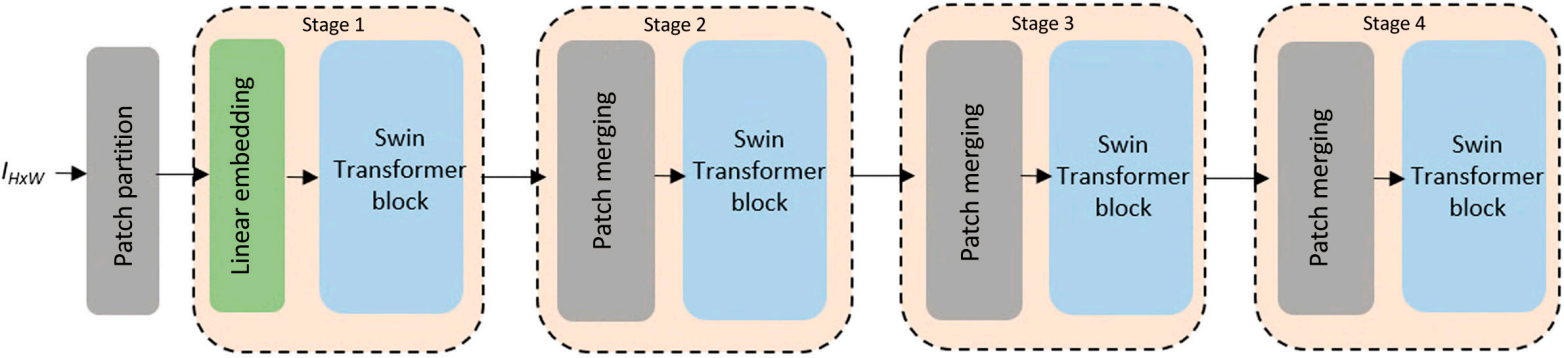
The architecture of Swin Transformer.

Swin Transformer builds a hierarchical feature map for the localization of an object during training. The idea of transformer-based DNNs was implemented in Natural language processing for several years. Subsequently, the vision transformer (ViT) concept was extended to computer vision. Swin Transformer feature extraction comprises a hierarchical feature map and shift window transformer. These 2 stages of hierarchical feature extraction in ViT improves the accuracy in detection and segmentation tasks. In ViT, a multi-head self-attention (MSA) module is used. It is replaced by a Swin Transformer block consisting of window MSA (W-MSA) and shifted window MSA (SW-MSA). The architecture of the Swin Transformer is depicted in Fig. 4

#### FRCNN-A: Faster-RCNN with ASpikeNet and attention module

In our previous research [1], we compared several spike detectors from 2 different phenotyping facilities. We observed that spikes emerging in the middle of the plant surrounded by leaves pose the most significant challenge for DNNs. FRCNN or other 1- and 2-stage detectors are mostly designed and applied to objects in the natural environment, such as crop disease detection or application in field phenotyping. In these cases, a foreground object’s relative size is larger than the background structures. The salience of spikes’ features in subsequent convolution layers gradually diminishes in DNNs as the network advances, leading to the loss of valuable information. To address this issue, we made several modifications to the architecture of FRCNN to reduce the dilution of essential features and localize a relatively small object, such as a spike pattern, a shallow feature extractor retained to reinforce the weight of the region of interest.

#### ASpikeNet

Similar to other DNNs, the input to the FRCNN-A network is of size 2,560 × 2,560. The image has been reduced to 2,560 × 2,560 by cropping it from the resolution of 3,315 × 4,462. The specific resolution was chosen to keep a multiple of 32 for the convolution layer, which can help optimize the network’s performance and memory usage. It is convolved with a 3 × 3 filter kernel in each layer. ASpikeNet utilizes a backbone architecture consisting of 5 convolution layers, where the filter combination employed is [12, 18, 36, 48, and 72]. The output resolution and feature map of the training images in convolution layers are summarized in Table 2.

**Table 2.** Overview of the FRCNN-A layer architecture

Layer name	Output resolution	Architecture
conv-1	1,280 × 1,280	3 × 3 × 12
conv-2	640 × 640	3 × 3 × 18
conv-3	320 × 320	3 × 3 × 36
conv-4	160 × 160	3 × 3 × 48
conv-5	80 × 80	3 × 3 × 72

The ASpikeNet is adapted by a 3 × 3 block that mirrors a residual block in ResNet [22]. We used the layers *CONV3*, *CONV4*, and *CONV5* as pyramid attention blocks and act as multilayer feature fusion. It is scaled by 1 × 1, 2 × 2, and 4 × 4. The higher layers extract more abstract and contextual relevant features for spikes. The layer CONV5 also recovers the features to their original size with bilinear interpolation. It combines the attention-weighted feature maps from different contextual levels of the feature pyramid. The feature fusion aims to produce a unified representation that captures local spike contours. The features from multiple scale are concatenated.

The extracted features to find the patches with the object are fed into RPN, which generates regions (proposals) of interest by predicting potential object bounding boxes. With only a mature spike in the training set, we choose a fixed anchor ratio. Instead of utilizing anchor boxes with various aspect ratios, we can use anchor boxes with a fixed anchor ratio. This means that the aspect ratio of the anchor box remains the same throughout the RPN. The RPN anchor ratio covers the range of spike length, which changes in spikes of both the phenotyping facilities. Then, ROI pooling helps to align and extract fixed-size feature maps.

**Fig. 5.**
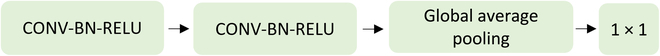
The elements of classifier in FRCNN-A.

In the classifier stage, the features extracted from the proposed regions are further processed to enable accurate classification and localization. The extracted features are input to 2 consecutive convolutional layers with batch normalization and rectified linear unit (RELU) activation functions (CONV-BN-RELU) followed by global average pooling and 1 × 1 convolution as illustrated in Fig. 5. This helps to enhance the discriminative characteristics of learned aggregated features. The global average pooling, a 1 × 1 convolution layer, performs linear transformations on features to get fine-grained adjustments and optimizations. The object function used in the classifier is softmax loss, measuring the discrepancy between the predicted class probabilities and the ground truth labels. The convolutional layer’s fused feature map is picked and passed through the global average pooling function. Based on those discriminative features, the classifier first predicts the existence of the detection target in the current patch, and the detector is followed to locate them accurately. The output from the detector is the probability of object, *p*, and bounding box regression $t^{j}=\left(t_{x}^{j},t_{y}^{j},t_{w}^{j},t_{h}^{j}\right)$. In the detector part, the loss implemented is smooth-L1.

### Evaluation metric in detection DNNs

mAP was used as the performance metric to evaluate the DNNs in this study. The mAP was computed as a weighted average of the precision values at different recall threshold levels. The precision was computed at 11 recall levels that were equally spaced between 0 and 1 to obtain the average precision value. *mAP*_0.5:0.95_ computes the average precision across a range of IoU thresholds, specifically from 0.5 to 0.95, with increments of 0.05. This comprehensive approach ensures a balanced evaluation of model performance, capturing both detection accuracy and localization precision.

In the context of the PASCAL VOC2007 evaluation measure, a mAP of 0.5 is achieved when the IoU between the predicted and ground truth bounding boxes is 0.5. The mAP metric offers a holistic perspective of the precision–recall curve, with the maximum precision value being determined for each recall level.

We have evaluated our dataset with commonly used metrics for object detection, such as PASCAL VOC and COCO detection measures. The mAP used to evaluate the localization and class confidence of spike is calculated as follows

$$\mathrm{mAP}=\frac{1}{N}\sum_{i=1}^{N}{\mathrm{AP}}_{i}$$ (1)

## Results

FRCNN-A and the other 2 DNNs were implemented under Python 3.8 on Ubuntu 20.04 OS, Ryzen 7 3800x, complemented by NVIDIA 2080 Ti graphics card as a training environment. The networks were optimized on an SGD optimizer. In our study, we trained 3 models for spike detection on the baseline dataset and *T*_1−9_. FRCNN required between 900 and 1,200 epochs, FRCNN-A required 800 to 1,000 epochs, and the Swin Transformer required 2,500 to 3,000 epochs, showcasing the varied epoch ranges necessary for optimal performance. The training and evaluation of the FRCNN-A mode were performed with images of wheat cultivars from 2 different phenotyping facilities, as described in Materials and Methods. While training our deep learning model, we employed a dynamic learning rate strategy to optimize model convergence. Specifically, we initialized the learning rate at 0.0001 for the initial training phase. At the midpoint of training, which corresponds to epoch 500 in our experimental setup, we applied a learning rate adjustment, reducing the learning rate by a factor of 0.1. On the 224 × 224 image size, the number of training parameters of FRCNN, FRCNN-A, and Swin Transformer amounts to 60 million, 54 million, and 50 million, respectively.

When evaluating the performance of different models based on average precision (AP), we observed that Swin Transformer achieved higher accuracy than FRCNN-A and FRCNN when applied to the original image dataset without any additional data transformation or augmentation (see Table 3 for detailed results).

**Table 3.** Summary of performance of DNNs on original image sets without augmentation. Values in boldface indicate the best-performing model.

DNNs	Backbone	Training set	*AP*_0.5_	*AP*_0.75_
FRCNN [11]	Inception v2	360	0.84	0.80
FRCNN-A	ASpikeNet	360	0.90	0.88
Swin Transformer [23]	ResNet101	360	**0.91**	**0.90**

**Fig. 6.**
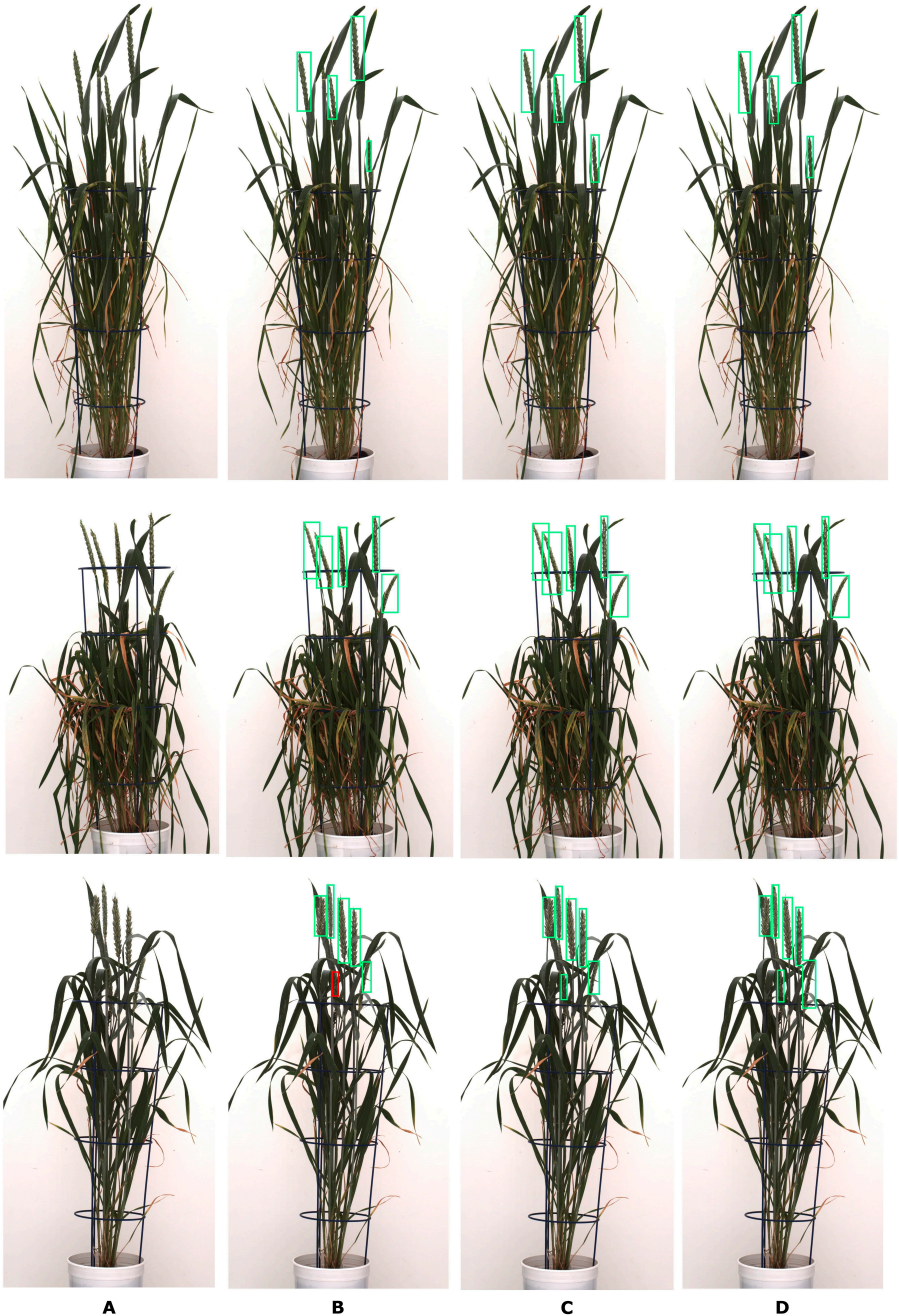
Detection of grain spikes in three different wheat cultivars (top, middle, bottom rows) using three different DNNs: (A) original image, (B) FRCNN, (C) FRCNN-A, and (D) Swin Transformer. Green bounding boxes indicate detected spikes, while red-framed spikes were not detected.

When comparing the performance of these 3 DNNs, FRCNN-A with attention module outperforms FRCNN by 6.89%, while Swin Transformer outperforms FRCNN-A by 1.1%. However, there is still a place for further improvement in FRCNN-A architecture, as evidenced by the higher accuracy of the Swin Transformer implemented as a dynamic window. The attention module captures the hierarchical context of ROI in multiple domain windows. The most challenging tasks for DNN detectors represent images with inside emerging within the mass of leaves. All 3 DNNs can detect top spikes with 100% accuracy. Table 4 summarizes evaluation tests carried out with spikes of different locations, including top emerging, inner spikes surrounded by leaves, and occluding spikes. Inner spikes were defined as ones that reside inside the plant canopy, including, in some instances, the spike lying at the periphery of the canopy. In that case, the criteria are if the spike is surrounded by more than half of the plant leaves, it is classified as an inner spike; otherwise, it is a top spike. The spike detection of the 3 DNNs—FRCNN, FRCNN-A, and Swin Transformer—is depicted in Fig. 6. Inner spikes are defined as the ones that reside inside the plant canopy. They may be partially or fully surrounded by plant leaves or branches in the background, while in some instances, they may remain unenclosed; otherwise, it is a top spike. The spike detection of the 3 DNNs—FRCNN, FRCNN-A, and Swin Transformer—is depicted in Fig. 6.

**Table 4.** Summary of detection model evaluation on matured top/inner spikes. Probability, *Pr*, is shown in the first column. *AP*_0.5_ and *AP_r_*:*AP*_0.5 : 0.95_ are PASCAL VOC and COCO evaluation measures, respectively. The numbers of the top and inner (occluding) spikes are 259 and 150. We have presented the results with higher accuracy by reporting the values up to 3 decimal places for *AP*_0.5_ and *AP_r_*:*AP*_0.5 : 0.95_. Values in boldface indicate the best-performing model.

	Top spikes: 259			Inner spikes: 150		
DNNs	*Pr*	*AP*_0.5_	*AP_r_*:*AP*_0.5:0.95_	*Pr*	*AP*_0.5_	*AP_r_*:*AP*_0.5:0.95_
FRCNN [11]	0.80	0.920	0.640	0.57	0.760	0.380
FRCNN-A	0.91	0.999	0.670	0.70	0.810	0.450
Swin Transformer [23]	0.91	0.999	**0.680**	0.72	0.830	**0.460**

Finally, we trained the model on 9 training datasets composed of images from the 2 phenotyping facilities, as described in the “Image data” section. The training sets differ based on the combination of IPK original and augmented images. This computational experiment was designed to find how the models adapted and learned from augmented images and performed solely on the IPK test set. The number of PSI images is kept the same in all the training sets. The training sets *T*_3_, *T*_6_, and *T*_9_ have the same number of augmented IPK images (300) to test whether the DNN’s accuracy is enhanced from increasing original IPK images. Therefore, we conclude that when comparing the accuracy of *T*_3_, *T*_6_, and *T*_9_ that have an identical count of IPK images, the mAP of *T*_3_ < *T*_6_ < *T*_9_, regardless of the DNNs used.

On training set *T*_3_, FRCNN-A has an *AP*_0.5_ of 79.11, and Swin Transformer has an increase of 1.18% from FRCNN-A (80.05) and FRCNN (78.9). In *T*_6_ and *T*_9_, FRCNN-A has an *AP*_0.5_ of 84.58 and 85.00 while Swin Transformer has an *AP*_0.5_ of 87.76 and 89.45, an increase of 3.75% and 5.23%. Additionally, the mAP of DNNs increases on testing across *T*_1_ to *T*_3_, *T*_4_ to *T*_6_, and *T*_7_ to *T*_9_ that was aimed to determine if increasing the number of IPK-augmented images enhances the DNN’s accuracy. In summary, the mAP of FRCNN < FRCNN-A < Swin Transformer on *T*_3_, *T*_6_, and *T*_9_. Table 5 outlines the mAP of 3 detection models, FRCNN, FRCNN-A, and Swin Transformer, trained on 9 different training sets and tested on the IPK test set.

**Table 5.**
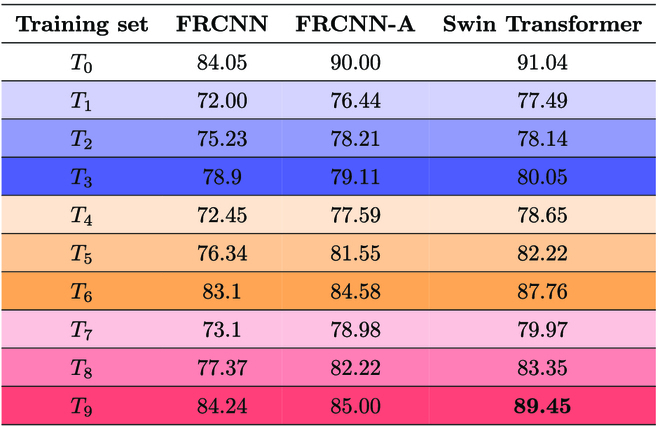
Summary of mAP of detection models trained on T0 and IPK-augmented 9 training sets (T1 to T9) and tested on the IPK test set. Similar to Table 1, the color code corresponds to the relative amount of IPK images: blue gradient for T1 to T3, orange gradient for T4 to T6, and red gradient for T7 to T9, respectively. The value in boldface indicates the best-performing model.

Comparing FRCNN-A and FRCNN, the modification introduced in the original FRCNN shows improvement in all the training sets. On *T*_6_ and *T*_9_, FRCNN-A increases *AP*_0.5_ by 1.78% and 0.90%. The average mAP of 3 DNNs on the training set *T*_0_ is 88.3% while the mAP on the training set *T*_9_ is 86.23%. The FRCNN parameters were set to default. The same activation function as that in FRCNN-A is used. In RPN, the anchor box is also kept fixed as FRCNN. Thus, most of the other part network models are unchanged. The advanced and adaptive architecture of the Swin Transformer included a hierarchical feature map and shifted window attention, which is highly mechanized for the localization of objects of different complexity. The additional attribute of the Swin Transformer is its highly adaptable extraction of feature maps on several scales. In a based network, this part of the setting is defined and customized for specific object types.

**Fig. 7.**
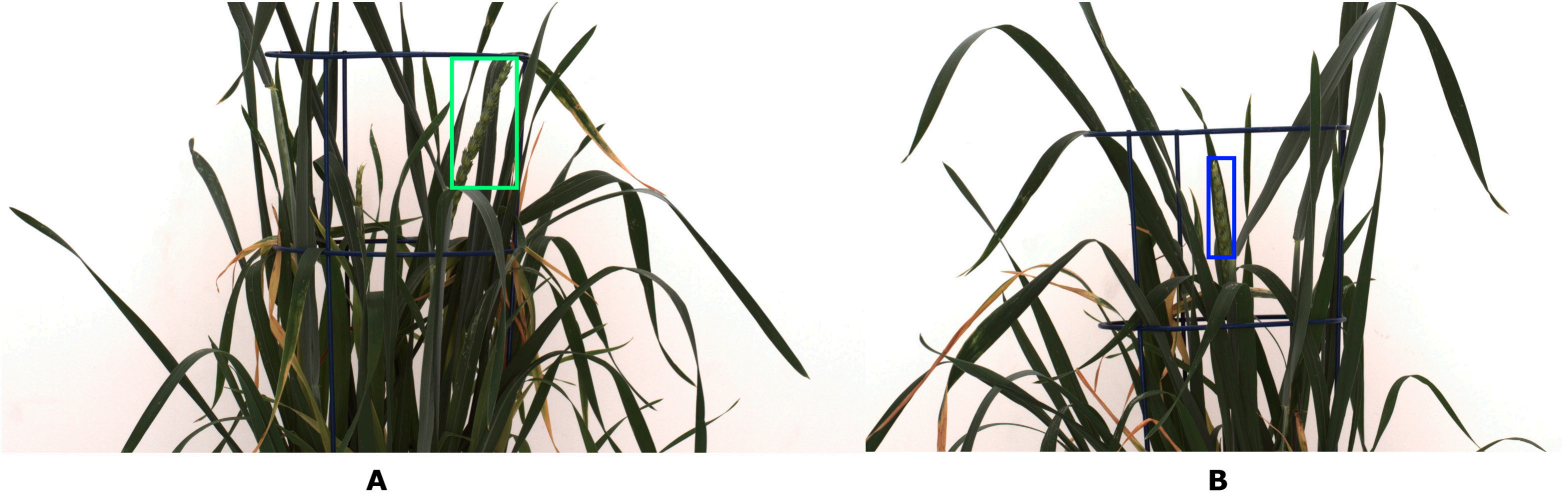
Detection of non-trivial test images: Swin Transformer: (A) detected inner spike, green box; and (B) failed detection of the inner spike, blue box.

The inner spike has been the most difficult to localize due to the lesser prominence of the spike contour in the feature extraction pipeline. Swin Transformer has the highest mAP; an example of the undetected spike is shown in Fig. 7B.

**Fig. 8.**
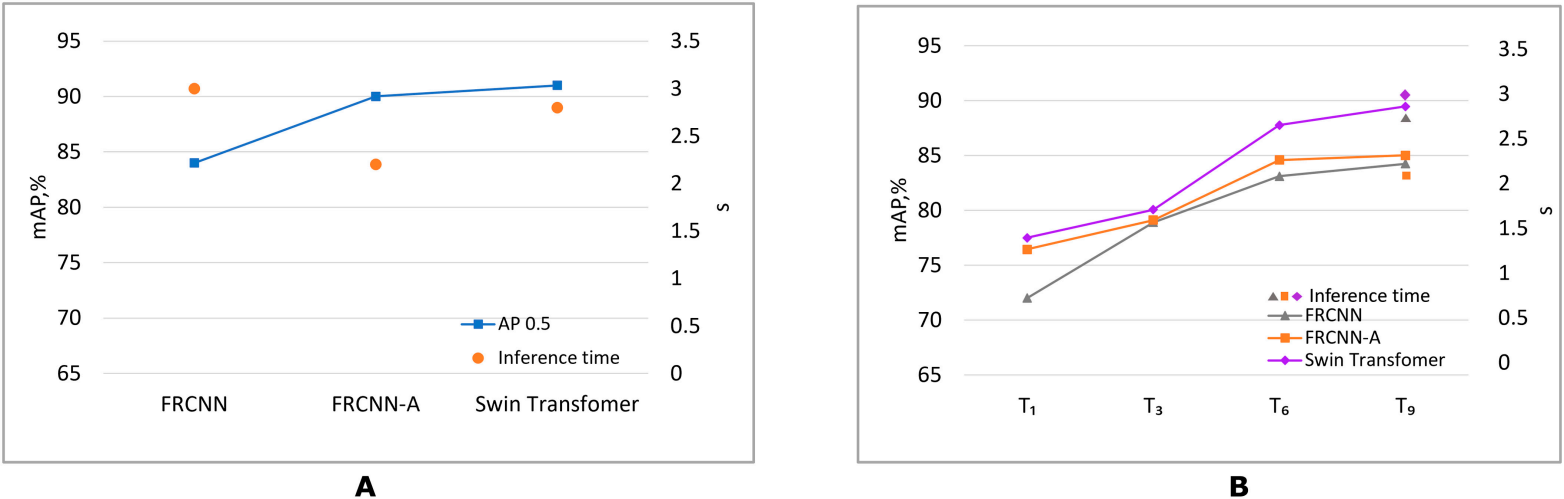
Accuracy and inference time. (A) The mAP and inference of DNNs on the original dataset (Table 3) and (B) the mAP and inference time of DNNs on the training set *T*_1_, *T*_3_,*T*_6_, and *T*_9_ from Table 4.

To evaluate the performance of the 3 networks, we measured their inference time on a test set of the original resolution using the trained model on the baseline and *T*_1−9_ training set. We ran our inference model on a similar computer used for training with a Ryzen 7 3800x CPU and an Nvidia GTX 2080 Ti GPU. The average inference time per image of FRCNN, FRCNN-A, and Swin Transformer is 3, 2.2, and 2.8 s on the original baseline dataset. In terms of inference time, FRCNN-A outperforms the state-of-the-art Swin Transformer model, offering enhanced speed and efficiency. The mAP and inference time of the 3 DNNs on original resolution and with the combination of the training set is shown in Fig. 8. The inference time is plotted against the training set 9 in Fig. 8B. Except for *T*_9_, which had the highest mAP and on average 0.05-s lower inference time for all the 3 DNNs, we observed no variation on average in the inference time of the model trained on other training sets (see Fig. 8).

## Discussion

In this study, we proposed several improvements to conventional FRCNN architecture to improve its robustness and accuracy for detecting relatively small and optically highly variable patterns of grain spikes in different wheat cultivars. To overcome the loss of feature information in successive convolution layers, we have reduced the number of convolution layers and performed the fusion of features from different scales, which helped to retain and enhance the core features of spikes. FRCNN has low recognition accuracy on a small object because 9 anchor boxes have equal aspect and area ratios, improving the position prediction. It performs well on relatively large objects in the PASCAL VOC dataset, while on spikes in mixed wheat cultivars composed of wheat cultivars from PSI and IPK, including augmented images, the accuracy of the detection network drops. The 3 DNNs’ results verified the effectiveness and robustness of the FRCNN-A proposed in this study. Swin Transformer and FRCNN-A have an attention mechanism to extract feature maps in their architectures. We see an improvement in detection accuracy in FRCNN-A on the non-augmented training set as the baseline case and also on augmented images in the training set. On the other hand, FRCNN suffers on non-augmented datasets with a mAP decrease of 6.67% and 7.70% compared to FRCNN-A and Swin Transformer. In Training sets *T*_3_, *T*_6_, and *T*_9_, the number of PSI and IPK augmented images is constant and only differentiated by the IPK original images to see its effect on the accuracy and generalization of DNNs. In our previous study [1], the trained network is generalized enough to detect spikes in unseen cereal crop datasets. In our study, the performance of FRCNN, FCNN-A, and Swin Transformer demonstrates noteworthy efficacy when evaluated on the *T*_3_, *T*_6_, and *T*_9_ training sets, which consist of a balanced composition of PSI and IPK augmented images with the difference of IPK original images. On *T*_3_, *T*_6_, and *T*_9_, all 3 DNNs exhibit the highest mAP relative to their prior training sets (*T*_3_ > *T*_1-2_, *T*_6_ > *T*_5−1_ and *T*_9_ > *T*_1-8_). Thus, the comparison of network performance shows a positive accuracy correlation with an increasing number of IPK original and augmented images, but when the composition is even between PSI and IPK augmented images, then the number of original IPK images in the training set enhances the mAP of all DNNs (9.6% on *T*_3_ to *T*_6_ and 1.92% on *T*_6_ to *T*_9_ in Swin Transformer). When comparing the DNNs based on the spatial position of spikes, the top spikes exhibit higher detection accuracy than the inner spikes, as shown in Table 4. The emerging spikes were not included in the current study. The inner spikes of the IPK test have less contrast to the leaves/stem in the background than PSI spikes. Therefore, they are more challenging to localize and extract. The training set of mixed cultivars also points to high generalization on both cultivars. The network tends to learn the features of each cultivar and adapt the feature map accordingly. In some test images, the Swin Transformer and other DNNs were unable to detect the non-trivial inner spike, as illustrated in Fig. 7.

The relevance of training of augmented images and fewer original images for instances in mAP of *T*_1_, *T*_4_, and *T*_7_ gives the clue of preservation of spike features in Gaussian noise, GAN-augmented and other transformation and networks tend to generalize features, and the retained features have similar to the non-augmented IPK images. The highest mAP achieved on *T*_9_ by DNNs, excluding FRCNN, is lower than when the network is trained on a baseline dataset The drop in AP is associated with the complexity of the feature map of the network when trained on mixed cultivars. The computational complexity and parameter training of the networks investigated in this study decrease from the Swin Transformer to FRCNN and FRCNN-A. FRCNN-A demonstrates reduced inference time relative to FRCNN and Swin Transformer across both the baseline and *T*_9_ training set. The inception v2 used to take a long time to train compared to our convolutional layers and attention module. The training convergence time of FRCNN-A is significantly shorter compared to both Swin Transformer and FRCNN.

Consequently, FRCNN-A presents a more efficient and faster training alternative, particularly for datasets that exhibit similar characteristics. Attention mechanisms generally improve the representation of model features by selectively focusing on the most prominent parts of the image. This can help to reduce noise and improve the accuracy of the model. Furthermore, by augmenting the IPK image and including its original IPK in the same training set, we aimed to investigate the generalization of the network. The presented solutions can be used to improve the detection of other small objects in large optical scenes, e.g., fruits, flowers, etc. Generalizing the trained network to detect spikes on limited original images of different wheat cultivars or other cereal crops remains challenging. Successive convolution layers in DNNs tend to lose detailed feature information. This issue is mitigated in our study by reducing the number of convolution layers, fusing features from different scales in FRCNN-A, or deploying a computationally intensive self-attention mechanism in Swin Transformer [complexity per layer: self-attention *O*(*n*^2^ · *d*) vs. recurrent *O*(*n* · *d*)]. However, there is a trade-off between inference time and accuracy, which, in the relatively higher-depth DNNs such as Swin Transformer, hinders its deployment in resource-constrained and real-time applications in embedded devices in greenhouse facilities. Although an ablation study of FRCNN-A was not carried out in this research, testing it on other benchmark datasets is planned in the future.

## Data Availability

Example images accompany this manuscript in Supplementary Materials. For other inquiries, please contact the corresponding authors.
